# Prenatal Diagnosis and Management of Generalized Arterial Calcification of Infancy

**DOI:** 10.1097/og9.0000000000000043

**Published:** 2024-11-07

**Authors:** Narmadha Kuppuswami, Boopathy Vijayaraghavan

**Affiliations:** Department of Obstetrics and Gynecology, Advocate Good Samaritan Hospital, Downers Grove, Illinois; and the Sonoscan Institute of Ultrasound in Medicine and the PSG Institute of Medical Sciences, Peelamedu, Coimbatore, Tamil Nadu, India.

## Abstract

**BACKGROUND:**

Generalized arterial calcification of infancy is a rare genetic disorder that was first reported in 1899. Since then, slightly more than 200 cases have been documented. This rare autosomal recessive disorder occurs when the homozygous *ENPP1* gene mutation is associated with abnormal calcification in the medium and large arteries in the fetus. Overall mortality in individuals with generalized arterial calcification of infancy is high (55%) before the age of 6 months, with 24.4% dying in utero. Due to the high mortality rate, there is a critical need for additional reports and research regarding generalized arterial calcification of infancy.

**CASE:**

We submit a case report of a child with generalized arterial calcification of infancy—a unique instance in which the diagnosis was established prenatally and treatment was initiated in utero. Patient care was continued through a multidisciplinary approach coordinated between specialists across India, Australia, and the United States. There was an almost complete resolution of the calcification, with near-normal development and intellectual capacity of the child for age.

**CONCLUSION:**

Clear guidelines are needed regarding prenatal use of bisphosphonate therapy, timing, dosage, and duration of treatment for fetuses with *ENPP1* gene mutations to minimize disease progression and improve neonatal health at birth. This case further illustrates how involving well-informed parents in decision making can help tailor the appropriate treatment. Our report also describes the possibility of new, ongoing clinical trials that may pave the way for a cure for this child and others.

## Abstract 2

Guidelines are needed regarding prenatal use of bisphosphonate therapy, timing, dosage, and duration of treatment to minimize disease progression when managing a fetus with an *ENPP1* gene mutation.

**Teaching Points**

• A negative ultrasound scan during the first trimester can give a false reassurance that the fetus is not affected by generalized arterial calcification of infancy.
• Unexplained low β-hCG and pregnancy-associated plasma protein A levels should raise a concern and need to be evaluated along with the family and personal history to assess the probability of recurrence risk.

We report the case of a second pregnancy in a 32-year-old patient whose fetus was diagnosed with generalized arterial calcification of infancy. Her first pregnancy was complicated by fetal growth restriction and resulted in an emergency cesarean delivery at 34 weeks of gestation. The neonate had severe hypertension due to generalized arterial calcification, with multi-organ failure and myocardial infarction and died after 4 days. Genetic evaluation revealed a pathogenic homozygous mutation in the *ENPP1* gene. Both parents were found to be carriers of the mutation, though they were not consanguineous.

## CASE

At 12 3/7 weeks of gestation in the second pregnancy, an ultrasonogram showed a nuchal translucency of 1.46 mm and triple test showed free β-hCG with 0.286 multiples of median and pregnancy-associated plasma protein A with 0.47 multiples of median. Because the patient's β-hCG and pregnancy-associated plasma protein A levels were low, she was advised to undergo amniocentesis or chorionic villus sampling but decided not to get tested and continued her pregnancy.

**Fig. 1.**
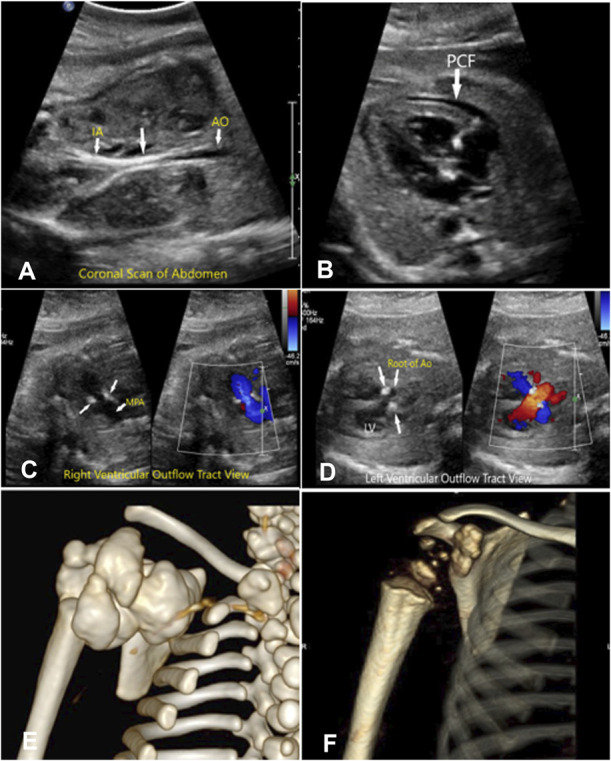
**A.** Coronal scan of the fetal abdomen shows the calcification (*arrows*) of the aorta (AO) and iliac (IA) arteries. **B.** Four-chamber view of the fetal heart showing pericardial effusion (PCF). **C.** Right ventricular outflow tract showing the calcification (*arrows*) of the main pulmonary artery (MPA). **D.** Left ventricular outflow tract showing the calcification (*arrows*) of the root of AO. **E.** Three-dimensional computed tomography image of the right shoulder at day 4. **F.** Three-dimensional computed tomography image of the right shoulder at age 20 months. LV, left ventricle.

An ultrasonogram at 29 weeks of gestation showed calcification in the distal one-third of the aorta, which was nonpulsatile (Fig. 1A). A fetal echocardiogram showed a moderate pericardial effusion (Fig. 1B) and calcification of the aortic and pulmonary valves extending into the pulmonary arteries (Fig. 1C and D), with normal cardiac activity and rhythm. The fetus was diagnosed with generalized arterial calcification of infancy.

*Kuppuswami. Management of Generalized Arterial Calcification of Infancy. O&G Open 2024*.

After consulting a pediatric endocrinologist, the patient was started on etidronate 20 mg/kg per day (1,600 mg) in two divided doses at 31.5 weeks of gestation. The working diagnosis was congenital generalized arterial calcification of the fetus. The patient underwent weekly monitoring of comprehensive metabolic panels and phosphate, vitamin D, 1,25(OH)2, parathyroid hormone, urine calcium, phosphate, and creatinine levels, along with weekly fetal ultrasound examinations. Her serum phosphate level increased to 2.84 mmol/L (normal 0.97–1.45 mmol/L), indicating an effect with etidronate. She also received vitamin D supplementation.

At 37 weeks of gestation, there was a significant reduction in the pericardial effusion and calcifications. However, the patient had no weight gain and the fetus showed poor fetal growth and had low amniotic fluid index; therefore, emergency cesarean delivery was performed for nonreassuring fetal evaluation. A 2,550-g neonate was delivered, with Apgar scores of 9 and 9 at 1 and 5 minutes. The neonate was vigorous and did not need resuscitation. Respiratory and cardiovascular functional parameters were normal. An echocardiogram showed a structurally normal heart; small patent foramen ovale; moderate patent ductus arteriosus; and echo brightness of the ascending and descending aorta, main pulmonary artery, and coronary arteries. A cranial ultrasonogram revealed mild calcification of the basal ganglia and perforate arteries. An abdominal Doppler ultrasonogram revealed thickened and echogenic aortic walls but unimpeded blood flow, mild patches of calcification in the renal artery with normal renal function, and normal blood pressure. A chest X-ray revealed extensive new bone formation at the level of the glenoid and the spine of the right scapula. The neonate also had periarticular calcification in her right shoulder, which limited the movement of her right arm (Fig. 1E). Radiographs of both wrists and knees showed no evidence of rickets. Results of an auditory brainstem response hearing test were normal. A skin biopsy revealed a pathogenic homozygous mutation in *ENPP1*.

The neonate was started on intravenous pamidronate infusions once per week for 4 weeks and then changed to oral risedronate 1 mg/kg weekly until age 7 months. At age 1 month, she was diagnosed with rickets, started on phosphate supplements, and registered at the National Institutes of Health generalized arterial calcification of infancy clinic. The family decided to stop bisphosphonate therapy at age 7 months. She continued to show improvement and eventually showed significant resolution of the calcifications by age 20 months. (Fig. 1F). Since November 2021, her dosage of phosphate supplementation has been doubled and she has been started on calcitriol 0.1 micrograms/day. Her teeth were poorly developed, with minimal enamel; therefore, she underwent molar buildup. She was diagnosed with autosomal recessive hypophosphatemic rickets, nephrocalcinosis, and unilateral craniosynostosis. She is currently on phosphate 750 mg in four divided doses, 0.4 micrograms of calcitriol in two divided doses, and 800 international units of cholecalciferol daily. Her developmental milestones are delayed, but she has normal intellectual capacity.

The patient has had two subsequent pregnancy terminations; one fetus was found to have a homozygous *ENPP1* gene mutation, and the other displayed no *ENPP1* mutation but was diagnosed with Down syndrome. She was counseled regarding the use of donor eggs for future pregnancies, but she declined.

## DISCUSSION

Generalized arterial calcification of infancy is a rare autosomal recessive genetic disorder that was first reported by Bryant and White in 1899.^1^ The onset of generalized arterial calcification of infancy during fetal development is characterized by abnormal calcification in the medium and large fetal arteries. In type 1, a homozygous mutation in the *ENPP1* gene leads to a deficiency in plasma inorganic pyrophosphate, promoting the widespread calcification of arteries.^2^ Type 2 (9–10%) is caused by mutations in the *ABCC6* gene and results in a condition called pseudoxanthoma elasticum that leads to ectopic mineralization most visible in the elastic tissues of the skin, eyes, and blood vessels.^3^ The incidence of this rare disorder is approximately 1 in 64,000 pregnancies (types 1 and 2 combined), with the East Asian population having a significantly higher carrier frequency than other populations^4^; the carrier prevalence is estimated at 1 in 200. Generalized arterial calcification of infancy affects males and females equally around the world.^2^ Patients with type 1 generalized arterial calcification of infancy are more likely to develop severe hypertension, cardiac failure, fetal distress, visceral effusions, coronary arterial occlusion, myocardial ischemia, and stenosis of different arteries, resulting in end-organ damage.^2^ In addition, they can develop extravascular periarticular calcifications, hearing loss, and rickets when they survive past the neonatal period.^2^

The calcification in the fetal arteries often is exhibited only in the second half of pregnancy. The regulation of mineralization by inorganic pyrophosphate and inorganic phosphate plays a crucial role in this process.^5^ It is known that inorganic pyrophosphate inhibits mineralization and inorganic phosphate stimulates it.^5^ The placenta produces alkaline phosphatase, which continues to increase throughout pregnancy and cleaves inorganic pyrophosphate to inorganic phosphate, resulting in an increase in inorganic phosphate^5^; homozygous *ENPP1* deficiency also results in a reduction in inorganic pyrophosphate. This drastic increase in inorganic phosphate in the second trimester is the reason for the appearance of calcification during the latter half of pregnancy.^5^ After delivery, loss of placental alkaline phosphatase leads to an increase in inorganic pyrophosphate. Furthermore, maturation of the fetal kidneys increases the clearance of inorganic phosphate. These factors may explain the postdelivery improvement in vascular calcification observed in some cases.^5^

No definitive treatment option exists for fetuses exhibiting the features of generalized arterial calcification of infancy. Bisphosphonates have been used in the treatment of generalized arterial calcification of infancy since the late 1970s and are the most widely used drug class, with varying degrees of success, but considerable controversy still exists. The recommended treatment options in the literature are to give etidronate as the first line of treatment and pamidronate and sodium thiosulfate as the second line if etidronate is unavailable.

The precise mechanism of action by which bisphosphonates influence outcomes in children with generalized arterial calcification of infancy is not known, but, by providing an alternate source of inorganic pyrophosphate, one can inhibit calcification. The therapeutic goal in generalized arterial calcification of infancy is to inhibit bone mineralization by using the less-potent etidronate. There are no studies available to determine the appropriate dose or duration of treatment. At present, only limited anecdotal data are available regarding the long-term use of bisphosphonate treatment during fetal development. At least one case of ulcerative esophagitis^6^ and another case of skeletal toxicity^7^ have been reported after long-term use of etidronate. The long skeletal half-life of bisphosphonates of up to 8 years after administration raises a major concern, especially for young children.^2,6–11^

Continued research is needed to determine effective prenatal and postnatal treatment methods. A study by Albright et al illustrates promising directions for research in which subcutaneous administration of ENPP1-Fc fusion protein reduced arterial calcification and mortality in a generalized arterial calcification of infancy mouse model.^12^ This has led to the first clinical trial (the ENERGY study) INZ701-104 to assess the safety and tolerability of INZ-701 in infants with *ENPP1* or *ABCC6* mutations.^13^ This child is already enrolled in this clinical trial. The results of this clinical trial may offer hope for families with children affected by these mutations. This case emphasizes the need for a clear understanding of the pathophysiology, the importance of genetic counseling, the parents’ comprehension of inheritance, a multidisciplinary approach to care, and accessibility to an ongoing clinical trial.
